# The Relationship of Serum Vitamin D Level With the Outcome in Surgical Intensive Care Unit Patients

**DOI:** 10.22037/ijpr.2019.1100658

**Published:** 2019

**Authors:** Sarvin Sanaie, Ata Mahmoodpoor, Hadi Hamishehkar, Shirin Fattahi, Saideh Soleymani, Elnaz Faramarzi

**Affiliations:** a *Tuberculosis and lung disease research center, Tabriz University of Medical Sciences, Tabriz, Iran.*; b *Faculty of Medicine, Tabriz University of Medical Sciences, Tabriz, Iran. *; c *Faculty of Pharmacy, Tabriz University of Medical Sciences, Tabriz, Iran. *; d *Faculty of dentistry, Tabriz University of Medical Sciences, Tabriz, Iran.*; e *Students research committee, Tabriz University of Medical Sciences, Tabriz, Iran.*; f *Liver and gastrointestinal diseases research center, Tabriz University of Medical Sciences, Tabriz, Iran.*

**Keywords:** Vitamin D, ICU, Sepsis, Mortality rate, Length of stay

## Abstract

The aim of this study was to assess the correlation of serum vitamin D with ICU length of stay, mortality rate, length of mechanical ventilation, and incidence of sepsis. We conducted a descriptive analytic study on 793 patients admitted to surgical ICU wards in northwest of Iran from March 2015 to March 2016. Patients were assessed during the ICU stay and the following data were collected: Glasgow Coma Score (GCS), APACHE II score, incidence of sepsis, duration of mechanical ventilation, LoS, mortality rate, and laboratory data (such as serum vitamin D, calcium, phosphorus, etc). The effect of vitamin D deficiency and the confounding factors on length of stay was assessed using the multinomial regression. Of 793 patients, 161 patients (20.3%) were in vitamin D deficiency group, 306 (38.6%) in vitamin D insufficiency group, 326 (41.1%) in vitamin D sufficiency group. Vitamin D deficiency increased risk of sepsis (OR = 22.93; 95%CI: 10.631-49.78) and mortality rate (OR = 42.93; 95%CI: 15.2-121.22). Vitamin D deficiency/insufficiency is a result of chronic and severe comorbidities of patients and can be considered as a helper but not a real risk factor for mortality and its level should be assessed in surgical critically ill patients. The way that various levels of vitamin D impact outcome in critically ill patients remains to be elucidated and further multi-center trials are needed to validate our results.

## Introduction

Despite the increased advances in critical care medicine, critically ill patients are still at high risk for mortality (1, 2). Some of the risk factors associated with mortality are modifiable ones and their identification and management can be an important way to improve outcome (3).

Vitamin D deficiency has a high prevalence in critically ill patients (4, 5) and some studies have shown an association between vitamin D deficiency and ICU outcomes (5-7). The vital role of vitamin D in calcium and phosphorous homeostasis is well recognized. Nowadays, other important roles have been determined for vitamin D such as modulating the innate and adaptive immune response to infectious pathogens, anti-inflammatory properties, and affecting cellular growth, proliferation and apoptosis (8-15). Therefore, vitamin D deficiency is associated with many diseases including certain cancers, immune system dysfunction, diabetes, cardiovascular disease, hypertension, lower respiratory infections, and metabolic syndrome (9, 16, 17). The prevalence of vitamin D deficiency has been reported to be between 40 to 80% among ICU patients (18-21).Taking into account the high prevalence of vitamin D insufficiency and deficiency and its important role in health status of humans, some studies have evaluated the association between serum vitamin D and outcome of critically ill patients. The findings of these studies indicated that vitamin D deficiency is correlated with disease severity and mortality, hospital costs, ICU length of stay, amount of time requiring mechanical ventilation, infection rates, bacteremia, multisystem organ failure, discharge location, and both short- and long-term mortality (22-29). However, this association has not been found in other studies (30-33) and the results of studies are conflicting. This observed discrepancy of the results may be due to the differences in sample size, method of measurements of vitamin D, and type of underlying disease. On the other hand, most of these observational studies have suggested to conduct further studies with large sample size to answer this question: “does vitamin D deficiency worsen the outcome of ICU patients?” Therefore, we decided to assess the correlation of serum vitamin D with the outcome in surgical ICU patients.

## Experimental

We conducted a descriptive analytic study on 793 patients admitted to surgical ICU of two university affiliated hospitals in northwest of Iran from March 2015 to March 2016, to assess the serum level of vitamin D and its relation with outcomes in this patient population. After obtaining approval from ethics committee of Tabriz University of Medical Sciences, the patients aging >18 years with completed informed consent were enrolled in this study. The patients with the history of intake of vitamin D, recent ICU admission (during the last month), and end stage cancer were excluded. 

The patients were assessed during the ICU stay and the following data were collected: age, sex, medical history, Glasgow Coma Score (GCS), APACHE II score, incidence of sepsis, duration of mechanical ventilation, ICU length of stay (LoS), mortality rate, and laboratory data including levels of serum vitamin D, calcium, phosphorus, urea, creatinine, albumin, hemoglobin and CRP. Vitamin D levels were measured in all patients during the first 24 hours of admission to the ICU with the chemiluminescence method. Based on the guidelines of the endocrine Society, vitamin D deficiency was defined as serum levels of 25(OH)D < 20 ng/mL, insufficiency as levels of 25(OH)D = 20–30 ng/mL, and sufficiency as 25(OH)D >30 ng/mL (21).

The primary outcome assessed in our study was ICU LoS. The secondary outcomes were mortality rate, length of mechanical ventilation, and incidence of sepsis. 

*Statistical Analysis*

The data were analyzed using Statistical Package for the Social Sciences (SPSS, version 11.5, Chicago, IL). We classified patients on the basis of vitamin D status into 3 groups (vitamin D deficiency, vitamin D insufficiency and vitamin D sufficiency). Descriptive statistics were obtained for all study variables and reported as mean ± SD and also number (percent) where applicable. One Way ANOVA test on quantitative parameters and the χ2 test on qualitative variables (gender, sepsis rate, GCS, mortality rate, duration of mechanical ventilation, APACHI Score) were used to determine whether these parameters differed between study groups. Multinomial regression was used to estimate Crude and adjusted odds ratios (OR) and their corresponding 95% confidence intervals (95% CI). ICU outcome (Sepsis rate, mortality rate, GCS, duration of mechanical ventilation) was considered as variable. Each variable was entered in the model one by one. The effect of confounding factors (age, serum calcium, phosphor, CRP, BUN, Cr, Hb, and Alb) were adjusted, and vitamin D sufficiency group was considered as a reference group. A P-value of less than 0.05 was considered statistically significant.

## Results

Demographic and clinical characteristics of patients stratified by vitamin D status are presented in Table 1. Of 793 patients, 161 patients (20.3%) were in vitamin D deficiency group, 306 (38.6%) in vitamin D insufficiency group, 326 (41.1%) in vitamin D sufficiency group. The results of one way ANOVA analysis indicated that clinical parameters of patients in vitamin D deficiency group significantly differ from the other two study groups (vitamin D insufficiency and sufficiency) (P < 0.05). The mean length of stay in hospital and duration of mechanical ventilation for vitamin D deficiency group were 24.13 ± 11.18 and 16.58 ± 9.04 days which were significantly higher than the other groups.

As shown in Table 2, the incidence of sepsis, low GCS (sever brain injury), mortality rate, and duration of mechanical ventilation in vitamin D deficiency group were more frequent than other groups. Most of the patients in vitamin D deficiency group had hospital LOS and mechanical ventilation more than 20 days while in other groups were less than 20 days. Moreover, according to APACHE score, the risk of death >40% were similar in the study groups.

**Table 1:** Demographic and clinical characteristics of patients stratified by vitamin D status

	**Vitamin D deficiency (n=161)**	**Vitamin D insufficiency (n=306)**	**Vitamin D sufficiency (n=326)**	***P*** **-value**
Age(year)	66.28±14.6	65.33±14.27	53.76±17.25	<0.001**
Male	67(41.6)	180(58.8)	255(78.2)	
Gender				<0.01***
Female	94(58.4)	126(41.2)	71(21.8)	
GCS	8.22±3.21	11.18±3.23	12.78±2.89	<0.001***
APACHE	26.30±4.72	22.45±4.11	20.38±3.14	<0.001***
Calcium(mg/dl)	7.80±0.47	8.01±0.39	8.18±0.47	<0.001***
Phosphorous(mg/dl)	3.44±0.44	3.58±0.41	3.67±0.37	<0.001***
Hb(g/dl)	11.14±1.09	11.55±1	12.02±1.28	0.01***
BUN	25.61±5.81	24.64±5.47	20.38±3.14	<0.001***
Cr	1.58±0.52	1.46±0.42	1.40±0.32	<0.001***
Alb(g/dl)	3.16±0.36	3.32±0.29	3.67±0.37	<0.001***
Dialysis	24 (14.9)	14(4.6)	8(2.5)	<0.001**
Hospital (LoS)*	24.13±11.18	12.26±7.91	6.21±4.52	<0.001***
Duration of mechanical ventilation(day)	16.58±9.04	9.02±5.98	7.10±11.27	<0001***

One Way ANOVA; Vitamin D deficiency (<20 ng/mL); Vitamin D insufficiency (20-30 ng/mL); Vitamin D sufficiency (>30 ng/mL).

* LoS: length of stay; #Number (percent),

* * P value: Comparison within group by χ2 test;

*** P value: Comparison within group by

**Table 2:** The prevalence of Sepsis, GCS, duration of mechanical ventilation according to vitamin D status

Vitamin D deficiency			Vitamin D insufficiency			Vitamin D sufficiency		
	**N**	**%**		**N**	**%**		**N**	**%**
Sepsis	59	36.66		56	18.3		8	2.5
**GCS**								
Sever (<8-9 )	95	59		72	23.5		38	11.7
Moderate (9-12)	47	29.2		113	36.9		66	20.2
Minor ≥13	19	11.8		119	38.9		221	67.8
In hospital mortality rate	56	34.8		11	3.6		4	1.2
Mechanical ventilation	156	96.2		191	62.4		83	25.5
**Duration of mechanical ventilation (day)**								
0-5	10	6.2		74	24.2		51	15.6
6-10	30	18.6		46	15		13	4
10-15	32	19.9		40	13.1		14	4.3
16-20	33	20.5		21	6.9		4	1.2
21-25	31	19.3		8	2.6			
26-30	5	3.1		2	0.7		-	-
31-35	9	5.6		-	-		-	-
36-40	4	2.5		-	-		-	-
41-45	2	1.2		-	-		-	-
**Hospital (LoS)** *								
1-10	14	8.7		150	49		278	85.3
11-20	48	29.8		97	31.7		44	13.5
21-30	55	34.2		52	17		-	
31-40	29	18		7	2.3		2	0.6
41-50	10	6.2		-			-	-
51-65	4	2.5		-			-	-
**APACHE Death risk**								
4	-	-		-			-	-
8	-	-		-			-	-
15	2	1.2		-			2	0.6
25	2	1.2		67	21.9		149	45.7
40	71	44.1		171	55.9		139	42.6
55	42	26.1		45	14.7		30	9.2
75	33	20.5		12	3.9		4	1.2
85	9	5.6		8	2.6		-	-

* LoS: length of stay; Vitamin D deficiency (<20 ng/mL); Vitamin D insufficiency (20-30 ng/mL); Vitamin D sufficiency (>30 ng/mL).

**Table3:** Predictor factors of ICU outcome

	**Vitamin D deficiency**				**Vitamin D insufficiency**				**Vitamin D sufficiency**			
	Unadjusted		Adjusted		Unadjusted		Adjusted		Unadjusted		Adjusted	
	OR (95% CI)	P	OR (95% CI)	P	OR (95% CI)	P	OR (95% CI)	P	OR (95% CI)	P	OR (95% CI)	P
Sepsis	22.93 (10.631-49.78)	<0.001	10.83(4.33-23.44)	<0.001	8.9(4.16-19.02)	<0.001	6.94(3.17-15.22)	<0.001	Ref			
GCS												
Sever (<8-9 )	29.07(15.99-53.03)	<0.001	26.92(13.53-53.57)	<0.001	3.51(2.24-5.52)	<0.001	3.4(2.1-5.49)	<0.001	Ref	-	Ref	-
Moderate (9-12)	8.28(4.54-15.08)	<0.001	6.92(3.43-13.97)	<0.001	3.18(2.18-4.63)	<0.001	3.25(2.14-4.93)	<0.001	Ref	-	Ref	-
Mortality	42.93(15.2-121.22)	<0.001	14.3(4.57-44.74)	<0.001	3(0.94-9.53)	0.06	1.5(0.41-5.01)	<0.001	Ref	-	Ref	-

Adjusted for serum Ca, P, Hb, BUN, Cr, CRP

Vitamin D deficiency (< 20 ng/mL); Vitamin D insufficiency (20-30 ng/mL); Vitamin D sufficiency (>30 ng/mL)

Predictor factors of ICU outcome are presented in table3. Vitamin D deficiency increased the risk of sepsis (OR = 22.93; 95%CI: 10.631-49.78 ) and mortality rate (OR = 42.93; 95%CI: 15.2-121.22) versus ( OR = 8.9; 95% CI:4.16-19.02) and (OR: 3; 95% CI: 0.94-9.53) in vitamin D insufficiency respectively. In addition, vitamin D deficiency were significantly correlated (OR = 29.07; 95% CI: 15.99-53.03) with low GCS <8-9. However, the correlation of vitamin D deficiency with ICU outcomes decreased after adjusting for age, serum calcium and phosphor, BUN, Cr, CRP, Hb, and Alb.

## Discussion

Our results showed that almost 57% of patients admitted to our surgical ICUs had vitamin D insufficiency/deficiency which was much higher rate compared to previous large trials showing prevalence between 20-40% (6, 20, 34). These results may be attributed to low economic status of our study population since they were selected from governmental hospitals. 

In addition, our samples have totally low exposure to sun light due to religious covering (hijab) and the geographical position of our region which is in north of Iran with high altitude. We showed a relation between 25 (OH) vitamin D deficiency and mortality. Similar to our findings, lee *et al*. showed a threefold mortality rate in vitamin D insufficient patients compared to patients with normal levels (5). Aygencel *et al.* showed that vitamin D deficiency is significantly associated with higher mortality rate but vitamin D deficiency is not an independent risk factor for mortality (32). Vitamin D levels were more significant in survivors compared to non-survivors. The explanation for the association of higher mortality rate with low levels of vitamin D may be due to the endothelial or immune cell dysfunction and impaired glucose and calcium metabolism accompanied with low levels of vitamin D. Contrary to previous trials, we didn’t find any significant correlation between serum levels of albumin and vitamin D deficiency (35) which may be due to the economic status and low levels of medical care of our patients. Risk factors for vitamin D deficiency are older age, diminished sunlight exposure, obesity, pigmentation and dark skin, low intake and malabsorption syndromes (19). Level of Vitamin D declined more during ICU stay because of insufficient replacement and absent sunlight exposure. We showed that vitamin D deficiency/insufficiency were more in older patients and females which is similar to previous trials (36). Higher prevalence of lower levels of vitamin D in females is related to religious and covering in our country and also could be a reflection of general vitamin D deficiency in our country. Our univariate analysis showed that vitamin D deficiency is associated with occurrence of sepsis which is similar to previous studies (37, 38). But after adjustment of different cofactors (Calcium, phosphorus, hemoglobin, blood urea nitrogen, creatinine and CPR) there was not a significant relation between sepsis and vitamin D levels which shows the possibility of being a cofactor, not a biomarker, for mortality in critical ill patients. A recently performed observational study showed that vitamin D deficiency is not associated with 90 day mortality in septic patients, but is associated with more hospital acquired infections and more acute kidney injury (39). We didn’t show any correlation between vitamin D levels and total calcium levels which could explain that the association of mortality and vitamin D levels is not modified by calcium levels. However, the best method for calcium assay in critically ill patients is measurement of ionized calcium not total calcium. Recently, Brook *et al* in a retrospective analysis showed that vitamin D level may be a modifiable risk factor for non-home discharge destination in surgical critically ill patients. Arnson in another study showed that vitamin D level in critically ill patients may be a marker of survival or a cofactor and they recommended assessment of vitamin D supplementation in critically ill patients (40). Ebenezer *et al* showed that Vitamin D deficiency is also common in critically ill pediatric patients admitted to ICU. Low serum 25 (OH) D level was associated with higher severity of illness, more days on mechanical ventilation, more vasopressor use and lower serum calcium levels. They couldn’t show any relation between vitamin D levels and mortality rate which may be due to the small sample size of their population (41). Anwar *et al.*, in their recently performed study in 250 critically ill patients, showed a high prevalence of vitamin D deficiency in these patients and showed its relation with severity of disease, but they could not confirm vitamin D as an independent risk factor for mortality in critically ill patients(42).

*Limitation*

First, we only analyzed surgical patients; so we cannot generalize these results to whole (surgical/medical) critically ill population. Second, we didn’t assay ionized calcium and PTH levels in our study and we could not exclude the confounding effects of these variables. Third, we did not determine vitamin D level sequentially; so its level on admission could be a reflection of preadmission deficiency as resuscitation and hemodilution can decrease the vitamin D levels. Thus, interpreting vitamin D levels in critically ill patients during resuscitation on ICU admission should be performed with caution (43). 

## Conclusion

Vitamin D deficiency is common in critically ill patients and causes different systemic effects in surgical ICU patients. Our study showed a strong association between hospital mortality and 25 (OH) vitamin D levels in critically ill patients. Vitamin D deficiency is associated with increase in length of ICU stay and sepsis. Vitamin D deficiency/insufficiency is a result of chronic and severe comorbidities of patients and can be considered as a helper but not a real risk factor for mortality and its level should be assessed in surgical critically ill patients. The way that various levels of vitamin D impact outcome in critically ill patients remains to be elucidated and further multi-center trials are needed to validate our results.
